# Global, regional, and national children and adolescent epilepsy burden, 1990–2021: an analysis based on the global burden of disease study 2021

**DOI:** 10.1080/07853890.2025.2534087

**Published:** 2025-07-17

**Authors:** Zhenjin Su, Jie Lu, Yuheng Shi, Bin Qi, Zeshang Guo

**Affiliations:** ^a^ Department of Neurosurgery, The First Hospital of Jilin University, Changchun, Jilin, China; ^b^ Department of Pediatric, The First Hospital of Jilin University, Changchun, Jilin, China

**Keywords:** Child and adolescent health, disease burden, epidemiology, epilepsy, public health

## Abstract

**Background:**

Epilepsy is a prevalent central nervous system disorder, affecting over 70 million people globally. It ranks fifth among neurological disorders in terms of disease burden. Epilepsy exhibits peak incidence in both childhood and older age, leading to a significant disease burden. This study aims to assess the burden of epilepsy in children and adolescents, analyze its key contributing factors, and identify strategies to mitigate the disease burden.

**Methods:**

The disease burden of childhood and adolescent epilepsy was analyzed across spatial, temporal, and population levels using data from the Global Burden of Disease 2021 study.

**Results:**

In 2021, over 8 million children and adolescents worldwide were living with epilepsy. The disease burden in this population is greater in males than in females, with morbidity primarily concentrated in children under 5 years of age and mortality predominantly occurring in adolescents aged 15–19. The current disease burden is strongly correlated with population and epidemiological factors, and while incidence and prevalence are projected to rise, both mortality and Disability-Adjusted Life Years (DALYs) are expected to decrease.

**Conclusion:**

This study highlights the global changes in the disease burden of epilepsy in children and adolescents. Population factors have been identified as the primary drivers of the increasing burden, while improvements in public health policies and access to treatments have contributed to the decreasing burden. Despite the expected rise in the future incidence, targeted improvements in treatment accessibility and health budget allocations are essential to reduce the long-term burden of epilepsy.

## Key Messages

1. Analyzed the burden of disease in children and adolescents with epilepsy in time, space, and population distribution.
2. Explored the burden of disease in different socio-demographic index regions.
3. Predicted trends in the disease burden of epilepsy in children and adolescents in 2050.

## Introduction

Epilepsy is a common chronic disorder of the central nervous system, and the reference definition of epilepsy is based on the International League Against Epilepsy (ILAE) guidelines for epidemiological studies. According to these guidelines, a case of epilepsy is defined as a person experiencing active, recurrent epileptic seizures (two or more) with no immediate cause and having had at least one seizure in the past 5 years, regardless of whether or not antiepileptic drug treatment is used [1]. Typical clinical manifestations include tonic–clonic seizures, dystonia, and disorientation [2]. Epilepsy contributes to approximately 1% of the global disease burden, affecting more than 70 million people worldwide, with over 2 million new cases diagnosed annually [3,4]. The disease code for epilepsy is ICD-9 [5]. About 50% of epilepsy cases have no known cause, though previous studies suggest associations with factors such as migraines, autoimmune disorders, and parasite infections [6–9]. In recent years, research has revealed molecular mechanisms of epilepsy, including mutations in CHD4, altered expression of certain inflammatory proteins, and changes in ion channels [8,10–15].

Globally, an estimated 3.4 billion people were affected by central nervous system (CNS) disorders in 2021, with epilepsy ranking fifth among all CNS disorders, following stroke, migraine, dementia, and encephalitis [5,16]. Although appropriate treatment can control seizures in 70% of patients, the fear of recurrence, brain tumors, and sudden death related to epilepsy significantly impacts the daily lives of affected individuals [5,17,18]. Meanwhile, Individuals with epilepsy are also at higher risk of maltreatment, neglect, and social marginalization [19]. The cumulative economic welfare loss associated with epilepsy was estimated at $647.37 billion, with economic losses for those under 20 years of age approaching $100 billion, and long-term mortality effects accounting for $152.55 billion [20]. Reducing the burden of neurological disorders is now a key target in the United Nations Sustainable Development Goals [21]. Epilepsy exhibits a bimodal distribution in terms of incidence, with peak occurrences in both childhood and older age [4,22,23]. In children, the recurrence rate of epilepsy is as high as 22%, contributing to a substantial disease burden [24]. Epilepsy is more prevalent in resource-poor regions, which also face national, societal, and other barriers that hinder proper recognition and management of the condition. The limited access to medical resources further exacerbates the disease burden in these areas, making it significantly higher than in more resource-rich regions [20,22].

The Global Burden of Disease (GBD) study collects and publishes data on diseases and injuries globally and by region/country, assessing the burden of 371 diseases. It is an important tool for evaluating the impact of diseases on population health worldwide [25]. This study uses data on the incidence, prevalence, mortality, and disability-adjusted life years (DALYs) of epilepsy in children and adolescents from 204 countries and 21 regions. The study analyzes the current disease burden through multiple models and predicts future changes in the burden of epilepsy.

## Method

### Data source

GBD 2021 (https://ghdx.healthdata.org/gbd-2021/sources) estimates the disease burden for 204 countries and regions from 1990 to 2021 across 371 diseases and injuries, with analyses by 21 regions and 25 subgroups, including age, sex, and other factors. The incidence and prevalence modeling of epilepsy in GBD 2021 was conducted using DisMod-MR 2.1 (Disease Modeling Meta-Regression, version 2.1). DisMod-MR 2.1 is a meta-regression tool that generates internally consistent estimates of prevalence, incidence, remission, and mortality by sex, region, year, and age group [25]. Years Lost Due to Disability (YLD) and Years of Life Lost (YLL) were summed to calculate the DALYs for different locations, age groups, sexes, years, and causes. GBD 2021 calculated the final estimates by simulating the model with 500 iterations. The final estimates represent the mean estimates of the 500 extractions, with 95% uncertainty intervals (UIs) represented by the 2.5th and 97.5th percentiles of the extractions [23,25]. The GBD project team anonymized all patient data used in the study. As a result, the University of Washington’s Institutional Review Board granted a waiver for informed consent and ethical review.

This study collected data on the incidence, prevalence, mortality, and DALYs of epilepsy in individuals aged 0–19 years from the GBD database using the GBD Results Tool (https://vizhub.healthdata.org/gbdresults/). The burden of epilepsy was analyzed across different SDI regions to assess the relationship between socio-demographic factors and the burden of epilepsy. The SDI is a composite indicator that includes three components: per capita lagged distributive income, the average level of education of the population aged 15 and over, and the total fertility rate for women under 25 years of age (TFU 25). The SDI value ranges from 0 to 1. Based on the SDI value of each country, countries are categorized into five groups: low SDI, low-middle SDI, middle SDI, high-middle SDI, and high SDI [2].

### Age standardization

Incidence, prevalence, mortality, and DALYs were collected for age groups <5 years, 5–9 years, 10–14 years, and 15–19 years. Age-standardized incidence, prevalence, mortality, and DALYs for each age group were calculated using the global standard population as defined by the Global Population Development Report (GPDR). This method eliminates errors due to age composition and enhances the reliability of the findings [4,23,25].

### Jointpoint regression and average annual percentage change

The Joinpoint regression model is a statistical method used to assess trends in the burden of disease over time. The model employs least squares regression to estimate the change patterns of indicators and identify turning points in the trend [2]. The average annual percentage change (AAPC) quantifies both the direction and magnitude of trend changes.

### Decomposition method

Decomposition analysis was performed using Das Gupta’s method to examine the impact of population age, epidemiological changes, and population size on the burden of epilepsy, with epidemiological changes referring to the shifts in epilepsy incidence, prevalence, and mortality between 1990 and 2021 [23].

### *The Bayesian age*–*period*–*cohort models*

Bayesian–age–period–cohort (BAPC) models are commonly used to predict future changes in the burden of disease for chronic diseases [26]. BAPC was applied to predict changes in the incidence, prevalence, mortality, and DALYs of epilepsy in children and adolescents by 2050.

### Statistical analysis

All statistical analyses were performed using R (version 4.3.3) and the Joinpoint Regression Program (version 4.9.1.0).

## Results

### Global trends

**Figure 1.**
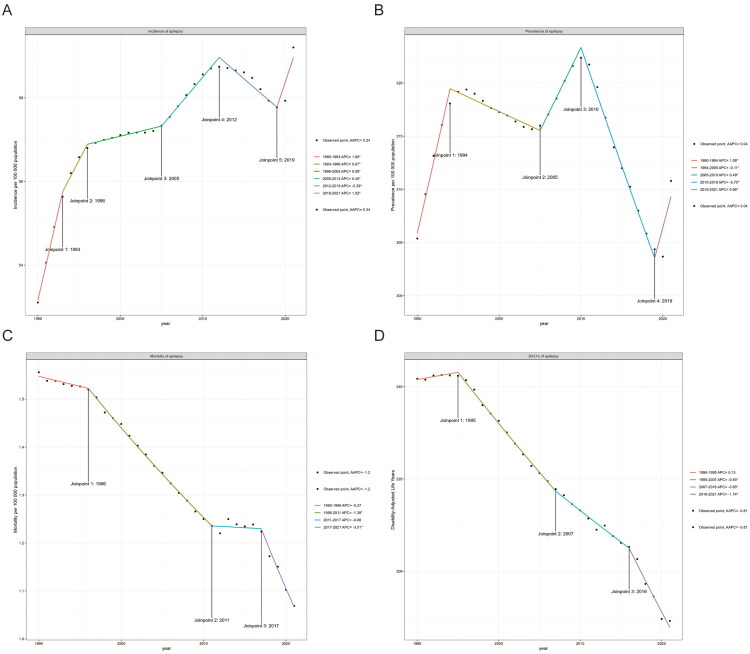
Global trends for age-standardized rates (per 100,000 population) of children and adolescent epilepsy from 1990 to 2021. (A) age-standardized incidence; (B) age-standardized prevalence; (C) age-standardized mortality; (D) age-standardized DALYs.

Figure 1 and Table 1 illustrate the changes in age-standardized incidence, prevalence, mortality, and DALYs of epilepsy in children and adolescents globally from 1990 to 2021. The incidence exhibited an overall increasing trend, rising from 53.1/100,000 (95% CI 31.4–81.5) in 1990 to 59.2/100,000 (95% CI 35.8–91.2) in 2021. The most significant increase occurred between 1990 and 1993. The incidence growth rate slowed after 1996. Between 2012 and 2019, there was a slight decline in incidence, from 58.7/100,000 (95% CI 35.5–89.0) in 2012 to 57.8/100,000 (95% CI 35.6–87.5) in 2019. The Incidence then increased again. The overall AAPC was 0.34 (95% CI 0.29–0.38). Prevalence fluctuated between 1990 and 2021 but did not show a significant overall change. The fastest rate of decline occurred between 2010 and 2019, from 322.4/100,000 (95% CI 218.9–451.1) to 304.4/100,000 (95% CI 210.1–424.4). The overall AAPC was 0.04 (95% CI 0.01–0.06).

**Table 1.** Incidence, prevalence, mortality and DALYs of children and adolescents epilepsy in 2021 and annual average percentage change in age-standardized rates.

Location	Incidence		Prevalence		Mortality		DALYs	
	2021 counts	AAPC in age-standardized rates, 1990–2021	2021 counts	AAPC in age-standardized rates, 1990–2021	2021 counts	AAPC in age-standardized rates, 1990–2021	2021 counts	AAPC in age-standardized rates, 1990–2021
Global	1550236 (933843 to 2396132)	0.34 (0.29 to 0.38)	8238420 (5530912 to 11681829)	0.04 (0.01 to 0.06)	28098 (21524 to 33862)	−1.2 (–1.3 to −1.09)	4984196 (3687505 to 6814410)	−0.81 (–0.87 to −0.74)
Low SDI	377966 (214551 to 587411)	0.12 (0.08 to 0.17)	1885621 (1208306 to 2770448)	−0.15 (–0.2 to −0.11)	10712 (7951 to 13605)	−1.06 (–1.3 to −0.83)	1591361 (1161062 to 2140538)	−0.84 (–0.93 to −0.75)
Low-middle SDI	433315 (255088 to 670512)	0.32 (0.25 to 0.4)	2393175 (1575314 to 3426583)	0.04 (–0.01 to 0.09)	10263 (7570 to 12639)	−1.15 (–1.41 to −0.89)	1675770 (1216173 to 2281065)	−0.8 (–0.91 to −0.7)
Middle SDI	426065 (247974 to 660680)	0.33 (0.28 to 0.39)	2313970 (1504809 to 3370828)	−0.02 (–0.08 to 0.04)	4826 (3836 to 5690)	−2.51 (–2.73 to −2.28)	1109461 (781706 to 1603358)	−1.49 (–1.58 to −1.4)
High-middle SDI	153810 (85200 to 250202)	0.35 (0.31 to 0.39)	834550 (516734 to 1254274)	−0.05 (–0.09 to −0.01)	1438 (1224 to 1719)	−3.11 (–3.52 to −2.71)	344187 (230221 to 520383)	−1.81 (–1.91 to −1.72)
High SDI	157855 (84916 to 254688)	0.3 (0.18 to 0.41)	804566 (481051 to 1229638)	0.22 (0.18 to 0.26)	841 (774 to 898)	−1.13 (–1.34 to −0.92)	259772 (155793 to 431752)	−0.46 (–0.56 to −0.37)
Andean Latin America	21919 (10222 to 37169)	0.01 (–0.2 to 0.22)	119249 (61108 to 193631)	−0.19 (–0.46 to 0.07)	168 (132 to 216)	−2.73 (–3.55 to −1.92)	49788 (28459 to 81267)	−1.5 (–1.73 to −1.28)
Australasia	4770 (1700 to 8720)	−0.09 (–0.13 to −0.05)	22198 (8394 to 37980)	−0.13 (–0.17 to −0.1)	26 (22 to 31)	−1.67 (–2.47 to −0.86)	7136 (3438 to 14246)	−0.95 (–1.27 to −0.63)
Caribbean	10770 (5649 to 17453)	−0.03 (–0.06 to 0.01)	55314 (30676 to 84816)	−0.14 (–0.18 to −0.11)	165 (115 to 240)	−0.73 (–0.91 to −0.55)	32309 (20685 to 47734)	−0.58 (–0.65 to −0.51)
Central Asia	24193 (12667 to 38234)	0 (–0.18 to 0.17)	146094 (84690 to 220126)	−0.1 (–0.18 to −0.01)	566 (457 to 693)	−0.16 (–0.44 to 0.13)	91963 (66533 to 125519)	−0.34 (–0.51 to −0.17)
Central Europe	14316 (7825 to 23197)	0.08 (0.06 to 0.1)	87246 (52799 to 132582)	−0.02 (–0.06 to 0.01)	126 (103 to 147)	−1.43 (–1.87 to −0.97)	32391 (21119 to 49186)	−0.95 (–1.15 to −0.74)
Central Latin America	77243 (44098 to 124469)	−0.2 (–0.23 to −0.18)	425044 (264963 to 630392)	−0.47 (–0.5 to −0.44)	823 (668 to 1014)	−1.46 (–2.15 to −0.76)	198498 (132863 to 294302)	−1.22 (–1.43 to −1.01)
Central Sub–Saharan Africa	57706 (21993 to 103746)	−0.11 (-0.26 to 0.03)	286430 (119030 to 495525)	−0.35 (–0.49 to −0.21)	1134 (748 to 1649)	−0.87 (–1.01 to −0.73)	203323 (119453 to 320791)	−0.77 (–0.9 to −0.65)
East Asia	127846 (69997 to 202287)	0.79 (0.65 to 0.94)	714479 (444332 to 1049411)	−0.02 (–0.08 to 0.04)	1870 (1490 to 2491)	−3.83 (–4.2 to −3.46)	350205 (244885 to 515284)	−2.67 (–2.95 to −2.39)
Eastern Europe	20312 (10812 to 32838)	−0.45 (–0.5 to −0.4)	99871 (58776 to 153501)	−0.51 (–0.55 to −0.47)	97 (82 to 110)	−3.24 (–4.19 to −2.29)	34575 (20129 to 56482)	−1.67 (–1.86 to −1.49)
Eastern Sub-Saharan Africa	173711 (96699 to 271925)	0.1 (0.06 to 0.14)	832353 (499712 to 1232406)	−0.03 (–0.09 to 0.02)	6376 (4878 to 8095)	−0.7 (–0.79 to −0.61)	830253 (616149 to 1112130)	−0.6 (–0.65 to −0.55)
High-income Asia Pacific	18747 (9669 to 30552)	0.29 (0.16 to 0.42)	92501 (51931 to 143060)	0.23 (0.12 to 0.34)	107 (95 to 120)	−1.26 (–1.59 to −0.94)	29847 (17439 to 51172)	−0.67 (–0.78 to −0.56)
High-income North America	49906 (25471 to 83338)	0.23 (0.18 to 0.28)	279941 (160868 to 433465)	0.12 (0 to 0.24)	254 (234 to 275)	0.48 (–0.26 to 1.23)	86807 (50096 to 148516)	0.02 (–0.1 to 0.14)
North Africa and Middle East	161223 (88860 to 260156)	0.16 (0.11 to 0.22)	858247 (539749 to 1292738)	−0.35 (–0.4 to −0.29)	2274 (1628 to 2834)	−2.13 (–2.34 to −1.92)	454844 (309406 to 667466)	−1.52 (–1.61 to −1.43)
Oceania	2549 (865 to 4838)	−0.04 (–0.06 to −0.01)	14742 (5469 to 26372)	0 (–0.06 to 0.07)	56 (35 to 87)	0.77 (0.62 to 0.92)	10222 (5605 to 17230)	0.2 (–0.01 to 0.41)
South Asia	296383 (170812 to 461566)	0.12 (0.03 to 0.21)	1792747 (1156058 to 2599495)	−0.2 (–0.32 to −0.08)	10215 (7454 to 12973)	−1.3 (–1.61 to −0.98)	1445145 (1052687 to 1933816)	−1.05 (–1.18 to −0.92)
Southeast Asia	119955 (66915 to 192750)	0.3 (0.25 to 0.34)	640215 (400303 to 956111)	0.36 (0.3 to 0.43)	518 (318 to 671)	−1.02 (–1.32 to −0.73)	266965 (162247 to 431199)	−0.25 (–0.3 to −0.19)
Southern Latin America	13439 (5422 to 24222)	0.39 (0.34 to 0.44)	64957 (28715 to 108631)	0.32 (0.26 to 0.39)	91 (78 to 106)	−0.82 (–1.57 to −0.06)	25232 (13680 to 44537)	−0.45 (–0.66 to −0.24)
Southern Sub-Saharan Africa	21409 (11854 to 34514)	−0.1 (–0.27 to 0.06)	108972 (63774 to 165158)	−0.23 (–0.37 to −0.09)	363 (279 to 486)	−0.02 (–0.46 to 0.42)	67825 (46558 to 96404)	−0.23 (–0.39 to −0.06)
Tropical Latin America	46024 (25626 to 72916)	−0.57 (–0.68 to −0.47)	235677 (143813 to 355034)	−0.8 (–0.9 to −0.69)	414 (332 to 492)	−0.45 (–0.99 to 0.09)	106897 (70126 to 162920)	−1.04 (–1.24 to −0.84)
Western Europe	74648 (39006 to 120658)	0.23 (0 to 0.46)	336480 (193864 to 517333)	0.17 (0.03 to 0.32)	355 (314 to 392)	−0.59 (–0.85 to −0.34)	105208 (61820 to 180858)	−0.3 (–0.47 to −0.13)
Western Sub-Saharan Africa	213166 (123284 to 328544)	0.1 (0.06 to 0.14)	1025663 (622572 to 1479966)	0.03 (–0.04 to 0.09)	2100 (1284 to 2739)	−0.09 (–0.25 to 0.08)	554765 (360648 to 820452)	−0.21 (–0.28 to −0.14)

Both mortality and DALYs showed a clear downward trend, decreasing from 1.6/100,000 (95% CI 1.1–1.9) and 241.7/100,000 (95% CI 176.7–318.8) in 1990 to 1.1/100,000 (95% CI 0.8–1.3) and 189.2/100,000 (95% CI 140.0–258.2) in 2021. Mortality and DALYs experienced the fastest rate of decline in 2017 and 2016, with AAPCs of −3.51 (95% CI −3.96 to −3.06) and −1.74 (95% CI −1.97 to −1.51), respectively. The AAPCs for mortality and DALYs from 1990 to 2021 were −1.2 (95% CI −1.3 to −1.09); and −0.81 (95% CI −0.87 to −0.74), respectively.

#### 5 SDI regional and geographic regional trends

**Figure 2.**
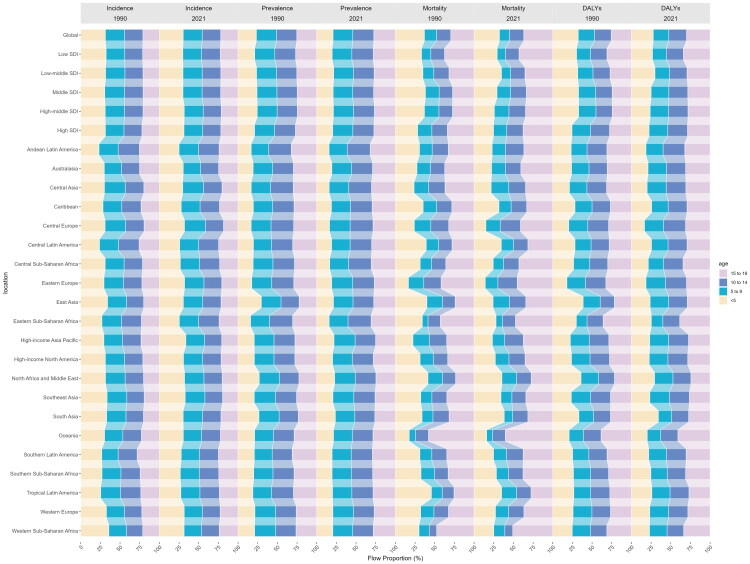
Age distribution of incidence, prevalence, mortality and DALYs for children and adolescent epilepsy by 21 regions, 1990 and 2019.

As shown in Figure 2, there were no significant changes in the age distribution of incidence, prevalence, mortality, and DALYs across regions between 1990 and 2021. In 2021, with the exception of Andean Latin America and Eastern Sub-Saharan Africa, the <5 years age group accounted for the highest proportion of epilepsy incidence within the 0–19 age range. The highest proportion was observed in the High-income Asia Pacific, where this age group represented 33.6% of all cases. In terms of prevalence, the 15–19 and 10–14-year age groups generally accounted for higher proportions among the 0–19-year-old population across most regions, except in the High-income Asia Pacific. Among the regions where the 15–19-year age group accounted for a relatively high proportion of epilepsy prevalence within the 0–19-year-old population, the Andean region of Latin America ranked first, with this age group representing 32.0% of all cases. Among the regions where the 10–14-year age group accounted for a relatively high proportion of epilepsy prevalence within the 0–19-year-old population, Eastern Sub-Saharan Africa ranked first, with this age group representing 29.4% of all cases. The age distribution of mortality varied considerably by region. However, most regions were dominated by 15–19-year olds, with a few regions having the highest proportion in the <5-year age group. Oceania ranked first, with this age group representing 60.3% of all cases. The age distribution of DALYs mirrored that of mortality rates.

**Figure 3.**
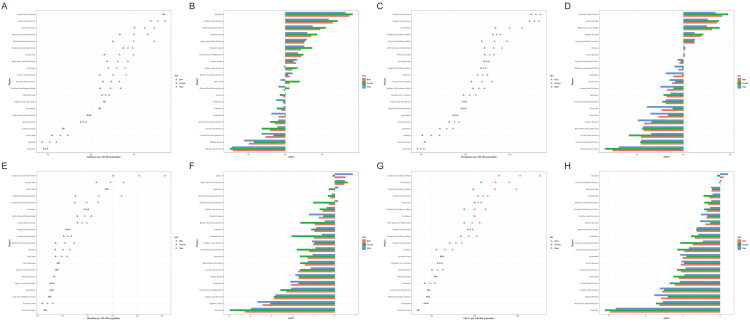
Regional age-standardized rates (per 100,000 population) of children and adolescent epilepsy in 2021 and their percentage changes in rates for different sexes from 1990 to 2021. (A) age-standardized incidence in 2021; (B) percentage change in age-standardized incidence 1990-2021; (C) age-standardized prevalence in 2021; (D) percentage change in age-standardized prevalence rate, 1990–2021; (E) age-standardized mortality in 2021; (F) percentage change in age-standardized mortality, 1990–2021; (G) age-standardized DALYs in 2021; (H) percentage change in age-standardized DALYs, 1990–2021.

The incidence of epilepsy in children and adolescents was higher in males than in females in all regions, except for high-income Asia Pacific and Andean Latin America. The highest incidence among the 21 regions was in Andean Latin America, at 92.6/100,000 (95% CI 43.2–157), while the region with the lowest incidence was East Asia, with 37.4/100,000 (95% CI 20.7–58.9) (Figure 3A). From 1990 to 2021, East Asia had the greatest increase in incidence, with an AAPC of 0.79 (95% CI 0.65–0.94), while the region with the greatest decrease was Tropical Latin America, with an AAPC of −0.57 (95% CI −0.68 to −0.47) (Figure 3B). Similar to incidence, prevalence was higher in males than in females most regions. The regions with the highest and lowest prevalence rates mirrored those with the highest and lowest incidence rates, namely Andean Latin America and East Asia, with prevalence rates of 499.4/100,000 (95% CI 255.9 to 811.1) and 205.8/100,000 (95% CI 128.6 to 301.4), respectively (Figure 3C). From 1990 to 2021, Southeast Asia had the largest increase in prevalence, with an AAPC of 0.36 (95% CI 0.3–0.43), while the region with the largest decrease was Tropical Latin America, with an AAPC of −0.8 (95% CI −0.9 to −0.69) (Figure 3D). Mortality and DALYs were higher in males than in females in most regions. The regions with the highest mortality and DALYs were Eastern Sub-Saharan Africa, at 2.8/100,000 (95% CI 2.16–3.58) and 366.4/100,000 (95% CI 272.1–490.7), while the regions with the lowest mortality and DALYs were Eastern Europe, with 0.20/100,000 (95% CI 0.17–0.23) and 73.6/100,000 (95% CI 43.0–119.9), respectively (Figure 3E,F). From 1990 to 2021, child and adolescent epilepsy mortality and DALYs increased only in Oceania and High-income North America, while they declined in all other regions. The region with the largest decline was East Asia, with AAPC of −3.83 (95% CI −4.2 to −3.46) and −2.67 (95% CI −2.95 to −2.39), respectively (Figure 3G,H).

#### 204 Country trends

**Figure 4.**
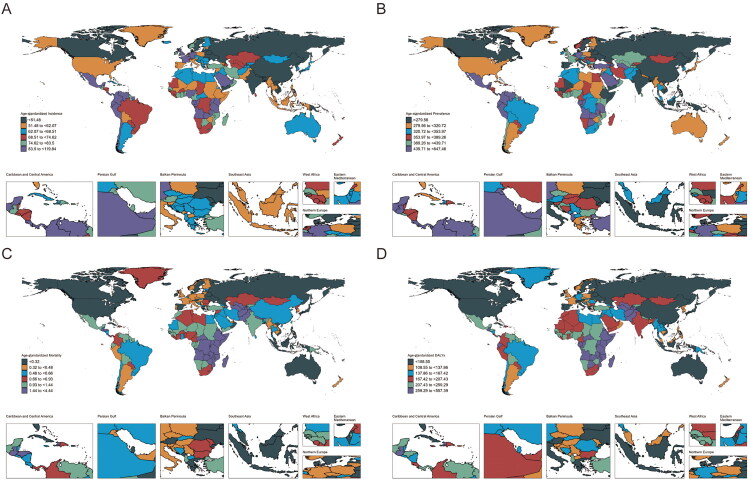
Countries’ age-standardized rates (per 100,000 population) of children and adolescent epilepsy in 2021. (A) age-standardized incidence; (B) age-standardized prevalence; (C) age-standardized mortality; (D) age-standardized DALYs.

Ecuador had the highest incidence at 119.8/100,000 population (95% CI 33.8–223.1), followed by Equatorial Guinea at 114.3/100,000 population (95% CI 29.7–216.9), while the lowest incidence was in the Democratic People’s Republic of Korea, at 30.8/100,000 population (95% CI 8.1 to 62.0). From 1990 to 2021, the incidence increased in 131 countries and decreased in 73. Equatorial Guinea had the largest increase, with an AAPC of 1.22 (95% CI 1.09–1.35), while Togo experienced the largest decrease, with an AAPC of −0.72 (95% CI −0.86 to −0.58) (Figure 4A; Table 2). Equatorial Guinea had the highest prevalence at 647.5/100,000 population (95% CI 159.4–1158.7), followed by Ecuador at 607.4/100,000 population (95% CI 189.3–1052.9). The lowest prevalence was in the Democratic People’s Republic of Korea, at 181.3/100,000 population (95% CI 47.9–332.6). From 1990 to 2021, prevalence increased in 101 countries and decreased in 103. Equatorial Guinea had the largest increase, with an AAPC of 1.41 (95% CI 1.29–1.52), while the largest decrease occurred in the Democratic People’s Republic of Korea. The largest decrease in prevalence occurred in the Democratic People’s Republic of Korea, with an AAPC of −1.24 (95% CI −1.31 to −1.17) (Figure 4B; Table 2). Zambia had the highest mortality rate at 4.4/100,000 population (95% CI 2.7–6.7), followed by South Sudan at 3.5/100,000 population (95% CI 2.2–5.1). The lowest mortality rate was in Viet Nam, at 0.03/100,000 population (95% CI 0.01–0.13). From 1990 to 2021, 35 countries saw an increase in mortality, while 169 experienced a decrease. Tokelau had the largest increase, with an AAPC of 4.69 (95% CI 3.74–5.65), while Latvia experienced the largest decrease, with an AAPC of −4.79 (95% CI −5.93 to −3.64) (Figure 4C; Table 2). Zambia had the highest DALYs at 557.4/100,000 people (95% CI 322.8–875.9), followed by the United Republic of Tanzania at 430.6/100,000 people (95% CI 245.7–685.4). The lowest DALYs were in the Russian Federation, at 67.2/100,000 people (95% CI 39.1–113.5). From 1990 to 2021, DALYs increased in 31 countries and decreased in 173. The largest increase occurred in Tokelau, with an AAPC of 2.06 (95% CI 1.03–3.09), while the largest decrease was in China, with an AAPC of −2.72 (95% CI −3.0 to −2.43) (Figure 4D; Table 2).

**Table 2.** Incidence, prevalence, mortality and DALYS for 204 country regions in 2021.

Location	Incidence (per 100 000 population)	Prevalence (per 100 000 population)	Mortality (per 100 000 population)	DALYs (per 100 000 population)
Afghanistan	−0.43 (–0.5 to −0.36)	−0.94 (–1 to −0.88)	−1.41 (–2 to −0.82)	−1.42 (–1.72 to −1.12)
Albania	0.32 (0.2 to 0.44)	−0.23 (–0.37 to −0.1)	−2.27 (–2.84 to −1.7)	−1.41 (–1.73 to −1.08)
Algeria	−0.25 (–0.33 to −0.17)	−0.81 (–0.86 to −0.76)	−2.29 (–2.48 to −2.1)	−1.87 (–1.93 to −1.81)
American Samoa	0.1 (–0.24 to 0.44)	0.16 (–0.03 to 0.35)	1.46 (0.05 to 2.9)	0.32 (–0.06 to 0.7)
Andorra	−0.2 (–0.26 to −0.14)	−0.29 (–0.34 to −0.25)	−2.66 (–3.1 to −2.21)	−1.15 (–1.41 to −0.9)
Angola	0.22 (0.12 to 0.31)	0.16 (0.08 to 0.23)	−1.05 (–1.41 to −0.69)	−0.63 (–0.82 to −0.44)
Antigua and Barbuda	0.07 (0.01 to 0.13)	0.03 (–0.02 to 0.07)	−0.56 (–1.72 to 0.61)	−0.44 (–0.76 to −0.12)
Argentina	0.64 (0.54 to 0.74)	0.74 (0.65 to 0.83)	−0.27 (–1.1 to 0.58)	0.19 (–0.05 to 0.44)
Armenia	−0.07 (–0.27 to 0.13)	−0.15 (–0.26 to −0.04)	−1.42 (–2.45 to −0.37)	−0.89 (–1.16 to −0.62)
Australia	−0.02 (–0.18 to 0.14)	−0.05 (–0.19 to 0.08)	−1.6 (–2.66 to −0.54)	−0.85 (–1.07 to −0.63)
Austria	0.12 (0.07 to 0.17)	0.11 (0.07 to 0.16)	−0.65 (–1.75 to 0.47)	−0.37 (–0.61 to −0.13)
Azerbaijan	0.25 (0.09 to 0.4)	−0.06 (–0.25 to 0.12)	−1.13 (–1.49 to −0.77)	−0.8 (–0.97 to −0.62)
Bahamas	−0.04 (–0.15 to 0.08)	−0.23 (–0.32 to −0.14)	−1.94 (–3.41 to −0.46)	−0.89 (–1.23 to −0.54)
Bahrain	0.07 (–0.02 to 0.16)	−0.38 (–0.48 to −0.28)	−2.32 (–2.78 to −1.86)	−1.69 (–1.82 to −1.57)
Bangladesh	0.1 (0.01 to 0.2)	0.02 (–0.12 to 0.16)	−2.56 (–2.95 to −2.17)	−1.65 (–1.91 to −1.38)
Barbados	−0.19 (–0.23 to −0.15)	−0.3 (–0.35 to −0.26)	−2.01 (–3.39 to −0.62)	−1.16 (–1.61 to −0.71)
Belarus	−0.6 (–0.71 to −0.49)	−0.67 (–0.73 to −0.6)	−3.15 (–4.05 to −2.23)	−1.94 (–2.24 to −1.65)
Belgium	0.14 (0.06 to 0.22)	0.09 (0.04 to 0.14)	−0.01 (–0.54 to 0.52)	−0.28 (–0.38 to −0.18)
Belize	0.24 (0.17 to 0.31)	−0.04 (–0.08 to 0.01)	−2.53 (–3.26 to −1.8)	−1.26 (–1.71 to −0.81)
Benin	−0.19 (–0.3 to −0.08)	−0.38 (–0.57 to −0.18)	−0.39 (–0.67 to −0.1)	−0.62 (–0.77 to −0.46)
Bermuda	−0.15 (–0.18 to −0.11)	−0.21 (–0.25 to −0.17)	−1.68 (–2.53 to −0.84)	−1.04 (–1.29 to −0.78)
Bhutan	0.23 (0.16 to 0.29)	0.34 (0.23 to 0.45)	−0.24 (–0.99 to 0.52)	−0.3 (–0.74 to 0.14)
Bolivia (Plurinational State of)	−0.56 (–0.69 to −0.43)	−0.97 (–1.1 to −0.84)	−2.53 (–2.59 to −2.48)	−2.03 (–2.08 to −1.97)
Bosnia and Herzegovina	0.25 (0.16 to 0.34)	0.15 (0.1 to 0.19)	−1.58 (–2.66 to −0.48)	−0.91 (–1.25 to −0.57)
Botswana	0.84 (0.73 to 0.96)	1.11 (1.04 to 1.17)	−0.32 (–0.6 to −0.05)	0.29 (0.17 to 0.41)
Brazil	−0.6 (–0.71 to −0.48)	−0.82 (–0.93 to −0.71)	−0.45 (–1.01 to 0.11)	−1.06 (–1.27 to −0.86)
Brunei Darussalam	−0.47 (–0.6 to −0.33)	−0.66 (–0.7 to −0.62)	−0.94 (–1.71 to −0.17)	−1.07 (–1.34 to −0.8)
Bulgaria	0.09 (–0.02 to 0.21)	0.02 (–0.05 to 0.09)	−0.61 (–1.45 to 0.23)	−0.5 (–0.83 to −0.16)
Burkina Faso	0.15 (0.08 to 0.22)	0.19 (0.1 to 0.28)	−0.19 (–0.38 to −0.01)	−0.09 (–0.19 to 0)
Burundi	−0.58 (–0.68 to −0.47)	−0.94 (–1.09 to −0.79)	−1.02 (–1.27 to −0.77)	−1.06 (–1.24 to −0.88)
Cabo Verde	0.72 (0.68 to 0.77)	0.5 (0.44 to 0.56)	−0.9 (–1.14 to −0.65)	−0.34 (–0.42 to −0.25)
Cambodia	0.22 (0.15 to 0.29)	0.14 (0.03 to 0.24)	−1.1 (–1.2 to −0.99)	−0.56 (–0.66 to −0.47)
Cameroon	0.06 (–0.05 to 0.16)	−0.03 (–0.13 to 0.08)	−0.32 (–0.64 to 0)	−0.32 (–0.42 to −0.22)
Canada	0.42 (0.3 to 0.54)	0.44 (0.29 to 0.58)	−0.94 (–1.92 to 0.06)	−0.23 (–0.51 to 0.04)
Central African Republic	−0.22 (–0.35 to −0.09)	−0.33 (–0.46 to −0.2)	0.1 (–0.08 to 0.29)	−0.11 (–0.21 to −0.01)
Chad	0.38 (0.34 to 0.41)	0.42 (0.3 to 0.53)	0.58 (0.29 to 0.87)	0.41 (0.34 to 0.48)
Chile	0.05 (0.01 to 0.09)	−0.11 (–0.16 to −0.06)	−1.28 (–1.81 to −0.75)	−1.1 (–1.2 to −0.99)
China	0.83 (0.68 to 0.98)	0.01 (–0.05 to 0.06)	−3.89 (–4.27 to −3.51)	−2.72 (–3 to −2.43)
Colombia	−0.18 (–0.21 to −0.14)	−0.46 (–0.51 to −0.41)	−1.95 (–3.4 to −0.47)	−1.47 (–2 to −0.93)
Comoros	0.15 (0.02 to 0.27)	−0.1 (–0.18 to −0.03)	−0.69 (–3.12 to 1.8)	−0.63 (–1.86 to 0.61)
Congo	0.02 (–0.04 to 0.08)	−0.13 (–0.15 to −0.1)	−0.68 (–1.19 to −0.16)	−0.64 (–0.83 to −0.45)
Cook Islands	0.15 (0.04 to 0.27)	0.1 (0.05 to 0.14)	−0.17 (–0.52 to 0.17)	−0.42 (–0.54 to −0.29)
Costa Rica	0.13 (0.03 to 0.23)	0.1 (0.05 to 0.16)	−1.02 (–1.99 to −0.03)	−0.6 (–0.85 to −0.35)
Coted’Ivoire	0.22 (0.12 to 0.32)	0.13 (0.06 to 0.2)	0.16 (–0.16 to 0.48)	−0.1 (–0.23 to 0.03)
Croatia	−0.17 (–0.24 to −0.09)	−0.28 (–0.33 to −0.23)	−1.25 (–2.25 to −0.23)	−0.92 (–1.33 to −0.51)
Cuba	−0.11 (–0.16 to −0.06)	−0.16 (–0.22 to −0.1)	−2.21 (–3.92 to −0.47)	−1 (–1.17 to −0.84)
Cyprus	0.07 (–0.02 to 0.16)	−0.03 (–0.12 to 0.07)	−3.41 (–4.52 to −2.29)	−1.28 (–1.62 to −0.94)
Czechia	−0.01 (–0.05 to 0.03)	−0.05 (–0.14 to 0.05)	−1.16 (–1.62 to −0.7)	−0.77 (–0.91 to −0.62)
Democratic People’s Republic of Korea	−0.3 (–0.45 to −0.14)	−1.24 (–1.31 to −1.17)	−1.44 (–1.52 to −1.36)	−1.58 (–1.73 to −1.42)
Democratic Republic of the Congo	−0.36 (–0.57 to −0.15)	−0.74 (–0.98 to −0.5)	−0.9 (–1.12 to −0.68)	−0.98 (–1.21 to −0.75)
Denmark	−0.54 (–0.61 to −0.46)	−0.7 (–0.8 to −0.6)	−0.25 (–0.94 to 0.45)	−0.97 (–1.1 to −0.83)
Djibouti	0.28 (0.25 to 0.3)	0.09 (0.03 to 0.14)	0.19 (–0.33 to 0.71)	−0.06 (–0.36 to 0.24)
Dominica	0.39 (0.34 to 0.44)	0.55 (0.51 to 0.59)	0.11 (–0.18 to 0.4)	0.24 (0.09 to 0.39)
Dominican Republic	0.48 (0.44 to 0.52)	0.36 (0.3 to 0.41)	−1.38 (–1.66 to −1.11)	−0.68 (–0.81 to −0.55)
Ecuador	0.33 (–0.02 to 0.69)	0.36 (–0.14 to 0.86)	−2.31 (–4.61 to 0.04)	−0.99 (–1.72 to −0.25)
Egypt	0.17 (–0.18 to 0.52)	−0.48 (–0.98 to 0.02)	−1.76 (–1.99 to −1.53)	−1.34 (–1.7 to −0.98)
El Salvador	0.25 (0.17 to 0.33)	0.01 (–0.18 to 0.2)	−2.4 (–2.58 to −2.21)	−1.33 (–1.6 to −1.06)
Equatorial Guinea	1.22 (1.09 to 1.35)	1.41 (1.29 to 1.52)	−2.12 (–2.51 to −1.73)	−0.47 (–0.69 to −0.26)
Eritrea	0.3 (0.1 to 0.5)	0.2 (0.07 to 0.34)	−0.18 (–0.47 to 0.11)	−0.12 (–0.39 to 0.15)
Estonia	0.29 (0.11 to 0.46)	0.08 (–0.11 to 0.27)	−3.86 (–4.71 to −3)	−2.29 (–2.55 to −2.02)
Eswatini	0.51 (0.48 to 0.54)	0.64 (0.59 to 0.68)	0.15 (–0.07 to 0.38)	0.3 (0.2 to 0.39)
Ethiopia	0.33 (0.19 to 0.46)	0.11 (–0.05 to 0.26)	−1.55 (–1.84 to −1.25)	−1.26 (–1.47 to −1.06)
Fiji	0.25 (0.14 to 0.37)	0.34 (0.27 to 0.41)	−0.01 (–0.35 to 0.33)	0.09 (–0.01 to 0.18)
Finland	0.4 (0.31 to 0.48)	0.43 (0.35 to 0.51)	0.16 (–0.33 to 0.64)	−0.02 (–0.41 to 0.38)
France	0.46 (0.36 to 0.56)	0.39 (0.31 to 0.47)	−0.39 (–0.8 to 0.03)	−0.26 (–0.54 to 0.01)
Gabon	0.16 (0.07 to 0.24)	−0.15 (–0.24 to −0.06)	−0.57 (–1.33 to 0.19)	−0.67 (–0.85 to −0.49)
Gambia	0.2 (0.16 to 0.24)	0.08 (0.02 to 0.13)	0.33 (–1.27 to 1.95)	−0.01 (–0.6 to 0.59)
Georgia	−0.22 (–0.47 to 0.04)	−0.89 (–1.07 to −0.7)	−1.97 (–2.85 to −1.08)	−1.38 (–1.93 to −0.83)
Germany	0.11 (0.02 to 0.19)	0.03 (–0.06 to 0.12)	−0.23 (–1.36 to 0.91)	−0.38 (–0.8 to 0.04)
Ghana	0.49 (0.41 to 0.57)	0.56 (0.44 to 0.67)	0.12 (–0.37 to 0.61)	0.21 (0.01 to 0.4)
Greece	0.05 (–0.03 to 0.14)	0.03 (–0.03 to 0.1)	1.25 (0.33 to 2.19)	0.07 (–0.25 to 0.39)
Greenland	−0.18 (–0.26 to −0.11)	−0.72 (–0.79 to −0.65)	−0.28 (–0.98 to 0.42)	−0.93 (–1.13 to −0.73)
Grenada	0.26 (0.2 to 0.32)	0.19 (0.11 to 0.27)	−2.05 (–3.32 to −0.76)	−0.93 (–1.32 to −0.53)
Guam	0.12 (0.07 to 0.18)	0.2 (0.15 to 0.24)	0.29 (–1.51 to 2.11)	0.02 (–0.26 to 0.29)
Guatemala	0.13 (0.07 to 0.19)	0.14 (0.06 to 0.22)	−1.54 (–1.82 to −1.26)	−1.16 (–1.53 to −0.78)
Guinea	0.14 (–0.06 to 0.33)	0.03 (–0.06 to 0.12)	−0.37 (–0.76 to 0.01)	−0.28 (–0.36 to −0.21)
Guinea–Bissau	−0.25 (–0.38 to −0.13)	−0.45 (–0.58 to −0.32)	−0.21 (–0.96 to 0.54)	−0.56 (–0.86 to −0.26)
Guyana	0.47 (0.42 to 0.51)	0.42 (0.38 to 0.46)	−0.74 (–2.21 to 0.74)	−0.23 (–0.84 to 0.39)
Haiti	−0.27 (–0.37 to −0.16)	−0.59 (–0.68 to −0.51)	−1.25 (–1.62 to −0.87)	−1.07 (–1.21 to −0.94)
Honduras	−0.47 (–0.53 to −0.41)	−0.85 (–1.03 to −0.67)	−1.9 (–2.03 to −1.78)	−1.57 (–1.73 to −1.41)
Hungary	−0.15 (–0.18 to −0.11)	−0.32 (–0.35 to −0.29)	−1.39 (–2.82 to 0.07)	−1.11 (–1.54 to −0.68)
Iceland	0.66 (0.57 to 0.75)	0.64 (0.57 to 0.71)	−0.05 (–1.88 to 1.81)	0.11 (–0.19 to 0.42)
India	0 (–0.12 to 0.12)	−0.32 (–0.48 to −0.16)	−1.58 (–2.01 to −1.15)	−1.3 (–1.49 to −1.1)
Indonesia	0.29 (0.21 to 0.36)	0.34 (0.25 to 0.42)	0.21 (–0.06 to 0.48)	0.02 (–0.08 to 0.12)
Iran (Islamic Republic of)	0.4 (0.31 to 0.48)	−0.45 (–0.53 to −0.36)	−3.62 (–3.86 to −3.38)	−2.21 (–2.37 to −2.05)
Iraq	0.36 (0.33 to 0.39)	0.12 (0.06 to 0.18)	−2.65 (–2.95 to −2.35)	−1.42 (–1.63 to −1.22)
Ireland	0.34 (0.3 to 0.38)	0.35 (0.33 to 0.37)	−1.16 (–1.84 to −0.47)	−0.39 (–0.54 to −0.24)
Israel	0.22 (0.11 to 0.34)	0.18 (0.02 to 0.34)	−0.84 (–1.48 to −0.2)	−0.43 (–0.65 to −0.21)
Italy	−0.22 (–0.55 to 0.11)	−0.25 (–0.52 to 0.03)	1.12 (0.77 to 1.47)	−0.31 (–0.61 to 0)
Jamaica	0 (–0.1 to 0.1)	−0.08 (–0.15 to 0)	−2.06 (–2.51 to −1.6)	−1.02 (–1.28 to −0.75)
Japan	0.51 (0.34 to 0.68)	0.6 (0.43 to 0.78)	1.16 (0.26 to 2.07)	0.48 (0.33 to 0.64)
Jordan	0.14 (0.09 to 0.18)	0.09 (0.02 to 0.16)	−2.78 (–2.91 to −2.64)	−1.42 (–1.6 to −1.23)
Kazakhstan	−0.05 (–0.22 to 0.11)	−0.03 (–0.2 to 0.14)	−0.17 (–0.97 to 0.64)	−0.37 (–0.62 to −0.12)
Kenya	−0.12 (–0.2 to −0.03)	−0.28 (–0.36 to −0.2)	−0.09 (–0.39 to 0.22)	−0.3 (–0.45 to −0.15)
Kiribati	−0.18 (–0.23 to −0.13)	−0.34 (–0.4 to −0.28)	−0.26 (–0.47 to −0.06)	−0.38 (–0.48 to −0.28)
Kuwait	−0.1 (–0.17 to −0.04)	−0.26 (–0.39 to −0.12)	−1.82 (–3.31 to −0.31)	−1.12 (–1.56 to −0.67)
Kyrgyzstan	−0.39 (–0.59 to −0.2)	−0.56 (–0.69 to −0.44)	−1.75 (–2.73 to −0.76)	−1.47 (–2.03 to −0.9)
Lao People’s Democratic Republic	0.25 (0.09 to 0.41)	0.26 (0.19 to 0.34)	−1.19 (–1.38 to −1)	−0.47 (–0.57 to −0.37)
Latvia	0.17 (0.07 to 0.27)	0.21 (0.12 to 0.31)	−4.79 (–5.93 to −3.64)	−1.99 (–2.48 to −1.5)
Lebanon	0.31 (0.27 to 0.35)	0.01 (–0.05 to 0.07)	−2.37 (–2.8 to −1.95)	−1.48 (–1.77 to −1.2)
Lesotho	0.67 (0.55 to 0.78)	0.82 (0.74 to 0.9)	0.95 (0.72 to 1.19)	0.85 (0.71 to 0.99)
Liberia	−0.51 (–0.64 to −0.38)	−1 (–1.19 to −0.81)	−1.69 (–2.19 to −1.2)	−1.52 (–1.74 to −1.3)
Libya	0.11 (0.04 to 0.18)	−0.54 (–0.59 to −0.49)	−1.38 (–1.68 to −1.07)	−1.22 (–1.39 to −1.04)
Lithuania	0.02 (–0.02 to 0.06)	0.04 (–0.02 to 0.1)	−3.18 (–3.99 to −2.37)	−1.64 (–2.14 to −1.14)
Luxembourg	0.08 (0.02 to 0.15)	−0.04 (–0.09 to 0)	−1.04 (–2.27 to 0.22)	−0.77 (–1.12 to −0.42)
Madagascar	−0.34 (–0.38 to −0.29)	−0.61 (–0.64 to −0.57)	−0.45 (–0.69 to −0.21)	−0.64 (–0.8 to −0.47)
Malawi	−0.11 (–0.2 to −0.01)	−0.34 (–0.39 to −0.28)	−0.76 (–1.2 to −0.31)	−0.7 (–1 to −0.4)
Malaysia	0.33 (0.31 to 0.36)	0.23 (0.18 to 0.28)	−1.86 (–2.05 to −1.67)	−0.6 (–0.75 to −0.44)
Maldives	0.02 (–0.03 to 0.07)	−0.24 (–0.29 to −0.19)	−1.7 (–2.53 to −0.86)	−1.2 (–1.42 to −0.98)
Mali	0.65 (0.4 to 0.9)	0.78 (0.48 to 1.08)	−0.24 (–0.71 to 0.22)	0.23 (–0.2 to 0.67)
Malta	0.35 (0.19 to 0.51)	0.38 (0.29 to 0.47)	1.68 (0.17 to 3.21)	0.24 (–0.03 to 0.5)
Marshall Islands	0.1 (0.07 to 0.12)	0.09 (0.03 to 0.15)	0.39 (0.23 to 0.56)	0.1 (–0.01 to 0.2)
Mauritania	0.01 (–0.1 to 0.11)	−0.09 (–0.19 to 0.01)	−0.76 (–1.08 to −0.43)	−0.7 (–1.01 to −0.38)
Mauritius	0.48 (0.43 to 0.52)	0.62 (0.56 to 0.69)	0.11 (–2.41 to 2.7)	0.19 (–0.85 to 1.25)
Mexico	−0.26 (–0.33 to −0.19)	−0.57 (–0.61 to −0.52)	−1.49 (–2.22 to −0.76)	−1.3 (–1.46 to −1.13)
Micronesia (Federated States of)	−0.09 (–0.16 to −0.02)	−0.23 (–0.27 to −0.19)	−0.59 (–0.66 to −0.52)	−0.54 (–0.59 to −0.49)
Monaco	0.07 (–0.02 to 0.16)	0.07 (0.01 to 0.13)	1.33 (1.11 to 1.55)	−0.07 (–0.18 to 0.04)
Mongolia	0.13 (0 to 0.25)	0.22 (0.18 to 0.27)	−2.66 (–3.82 to −1.5)	−1.52 (–1.96 to −1.07)
Montenegro	0.01 (–0.06 to 0.07)	−0.17 (–0.18 to −0.15)	−1.5 (–2 to −0.99)	−0.88 (–1.01 to −0.74)
Morocco	0.14 (0.1 to 0.18)	0.12 (–0.02 to 0.27)	−1.91 (–2.2 to −1.63)	−1.27 (–1.46 to −1.08)
Mozambique	0.5 (0.33 to 0.66)	0.49 (0.29 to 0.7)	−0.95 (–1.59 to −0.3)	−0.53 (–0.92 to −0.15)
Myanmar	0.2 (0.11 to 0.29)	0.23 (0.12 to 0.35)	−1.01 (–1.3 to −0.72)	−0.4 (–0.46 to −0.35)
Namibia	0.3 (0.26 to 0.34)	0.42 (0.39 to 0.45)	0.08 (–0.38 to 0.54)	0.02 (–0.15 to 0.19)
Nauru	−0.12 (–0.27 to 0.03)	−0.29 (–0.43 to −0.15)	−0.03 (–0.12 to 0.06)	−0.29 (–0.33 to −0.25)
Nepal	0.33 (0.24 to 0.42)	−0.4 (–0.51 to −0.3)	−1.85 (–2.06 to −1.63)	−1.5 (–1.68 to −1.32)
Netherlands	1.16 (1 to 1.32)	1.01 (0.79 to 1.23)	−0.23 (–0.99 to 0.54)	0.38 (–0.19 to 0.95)
New Zealand	−0.48 (–0.53 to −0.43)	−0.56 (–0.64 to −0.49)	−2.09 (–2.63 to −1.55)	−1.41 (–1.6 to −1.22)
Nicaragua	−0.1 (–0.12 to −0.08)	−0.55 (–0.6 to −0.51)	−3.11 (–3.24 to −2.97)	−1.9 (–1.97 to −1.82)
Niger	−0.28 (–0.31 to −0.25)	−0.39 (–0.44 to −0.34)	−0.55 (–0.95 to −0.15)	−0.62 (–0.87 to −0.37)
Nigeria	0.15 (0.05 to 0.26)	0.1 (–0.02 to 0.22)	0.06 (–0.14 to 0.26)	−0.15 (–0.24 to −0.06)
Niue	0.31 (0.22 to 0.39)	0.36 (0.3 to 0.42)	3.1 (2.64 to 3.55)	1.15 (0.77 to 1.54)
North Macedonia	−0.15 (–0.2 to −0.09)	−0.29 (–0.33 to −0.25)	−2.12 (–2.67 to −1.56)	−1.29 (–1.47 to −1.12)
Northern Mariana Islands	−0.08 (–0.28 to 0.12)	−0.07 (–0.2 to 0.05)	2.18 (0.66 to 3.73)	−0.1 (–0.29 to 0.1)
Norway	−0.03 (–0.11 to 0.06)	−0.06 (–0.13 to 0.01)	−2.23 (–3.09 to −1.36)	−1.01 (–1.37 to −0.65)
Oman	0.65 (0.61 to 0.69)	0.62 (0.57 to 0.68)	−1.87 (–2.32 to −1.41)	−0.47 (–0.6 to −0.33)
Pakistan	0.26 (0.18 to 0.34)	0.15 (–0.07 to 0.37)	−0.19 (–0.4 to 0.03)	−0.18 (–0.42 to 0.06)
Palau	0.03 (0.01 to 0.06)	−0.2 (–0.25 to −0.15)	−0.46 (–0.57 to −0.34)	−0.46 (–0.54 to −0.38)
Palestine	0.19 (0.16 to 0.21)	−0.02 (–0.06 to 0.03)	−2.13 (–2.62 to −1.64)	−1.4 (–1.71 to −1.09)
Panama	0.55 (0.51 to 0.59)	0.65 (0.58 to 0.72)	−1.34 (–1.69 to −0.97)	−0.36 (–0.51 to −0.21)
Papua New Guinea	0.04 (0 to 0.08)	0.1 (–0.02 to 0.23)	1 (0.81 to 1.19)	0.34 (0.26 to 0.42)
Paraguay	0.18 (0.12 to 0.25)	0.03 (–0.03 to 0.09)	−0.39 (–0.8 to 0.02)	−0.4 (–0.51 to −0.29)
Peru	0 (–0.14 to 0.13)	−0.26 (–0.46 to −0.07)	−3.44 (–4 to −2.89)	−1.76 (–1.96 to −1.55)
Philippines	−0.04 (–0.13 to 0.06)	−0.01 (–0.07 to 0.04)	−0.58 (–1.09 to −0.06)	−0.25 (–0.33 to −0.17)
Poland	0.46 (0.44 to 0.48)	0.59 (0.53 to 0.66)	−0.03 (–0.44 to 0.39)	−0.12 (–0.29 to 0.06)
Portugal	0.37 (0.27 to 0.48)	0.16 (0.06 to 0.26)	−1.38 (–2.24 to −0.52)	−0.87 (–1.33 to −0.4)
Puerto Rico	−0.02 (–0.11 to 0.07)	−0.03 (–0.1 to 0.04)	−3.95 (–4.69 to −3.22)	−1.3 (–1.45 to −1.16)
Qatar	−0.19 (–0.23 to −0.15)	−0.69 (–0.72 to −0.66)	−3.54 (–3.94 to −3.12)	−1.95 (–2.09 to −1.8)
Republic of Korea	−0.17 (–0.22 to −0.13)	−0.3 (–0.37 to −0.23)	−2.96 (–3.55 to −2.37)	−1.9 (–2.04 to −1.75)
Republic of Moldova	−0.5 (–0.56 to −0.43)	−0.56 (–0.62 to −0.5)	−2.65 (–3.5 to −1.79)	−2 (–2.39 to −1.61)
Romania	−0.12 (–0.16 to −0.07)	−0.18 (–0.31 to −0.05)	−1.56 (–2.52 to −0.6)	−1.24 (–1.64 to −0.85)
Russian Federation	−0.41 (–0.45 to −0.37)	−0.47 (–0.52 to −0.42)	−3.57 (–4.63 to −2.49)	−1.55 (–1.99 to −1.12)
Rwanda	−0.2 (–0.24 to −0.17)	−0.41 (–0.53 to −0.29)	−1.34 (–2.08 to −0.59)	−1.21 (–1.74 to −0.68)
Saint Kitts and Nevis	−0.05 (–0.16 to 0.05)	−0.24 (–0.35 to −0.14)	−1.33 (–3.02 to 0.38)	−1.11 (–1.38 to −0.84)
Saint Lucia	−0.03 (–0.05 to −0.01)	−0.25 (–0.34 to −0.16)	−1.56 (–2.1 to −1.02)	−0.97 (–1.15 to −0.8)
Saint Vincent and the Grenadines	0.23 (0.11 to 0.35)	0.09 (0.05 to 0.12)	−0.84 (–2.06 to 0.4)	−0.65 (–0.95 to −0.35)
Samoa	0.15 (0.11 to 0.2)	0.08 (0.04 to 0.12)	−0.84 (–0.98 to −0.7)	−0.34 (–0.41 to −0.27)
San Marino	0.05 (–0.04 to 0.14)	0.03 (–0.07 to 0.14)	−2.14 (–2.39 to −1.9)	−0.35 (–0.56 to −0.13)
Sao Tome and Principe	0.53 (0.5 to 0.56)	0.44 (0.37 to 0.51)	−0.38 (–1.99 to 1.25)	−0.23 (–0.71 to 0.25)
Saudi Arabia	0.65 (0.61 to 0.7)	0.55 (0.48 to 0.61)	−3.02 (–3.15 to −2.9)	−1.23 (–1.33 to −1.13)
Senegal	−0.03 (–0.11 to 0.06)	−0.08 (–0.18 to 0.02)	−0.04 (–0.9 to 0.82)	−0.31 (–0.68 to 0.05)
Serbia	−0.42 (–0.48 to −0.35)	−0.91 (–0.99 to −0.84)	−2.48 (–3.61 to −1.34)	−1.95 (–2.49 to −1.41)
Seychelles	0.39 (0.21 to 0.57)	0.43 (0.24 to 0.62)	−0.79 (–1.56 to −0.01)	−0.11 (–0.23 to 0.01)
Sierra Leone	−0.11 (–0.15 to −0.08)	−0.2 (–0.26 to −0.13)	−0.4 (–0.99 to 0.18)	−0.51 (–0.73 to −0.29)
Singapore	0.39 (0.33 to 0.44)	0.37 (0.34 to 0.4)	0.23 (–0.38 to 0.84)	−0.28 (–0.46 to −0.09)
Slovakia	0.19 (0.13 to 0.25)	0.19 (0.11 to 0.27)	−1.2 (–1.7 to −0.69)	−0.68 (–0.75 to −0.61)
Slovenia	−0.09 (–0.13 to −0.06)	−0.19 (–0.21 to −0.17)	−1.34 (–1.89 to −0.79)	−0.98 (–1.32 to −0.64)
Solomon Islands	0.19 (0.09 to 0.29)	0.06 (–0.07 to 0.2)	0.24 (–0.18 to 0.68)	0.04 (–0.28 to 0.35)
Somalia	−0.24 (–0.32 to −0.16)	−0.43 (–0.48 to −0.39)	−0.02 (–0.38 to 0.34)	−0.24 (–0.47 to −0.01)
South Africa	−0.23 (–0.48 to 0.02)	−0.34 (–0.57 to −0.11)	−0.6 (–0.86 to −0.33)	−0.66 (–0.76 to −0.56)
South Sudan	−0.33 (–0.47 to −0.19)	−0.69 (–0.75 to −0.63)	0.79 (0.07 to 1.52)	0.04 (–0.37 to 0.46)
Spain	0.01 (–0.22 to 0.23)	−0.05 (–0.24 to 0.14)	−0.49 (–0.89 to −0.09)	−0.44 (–0.65 to −0.23)
Sri Lanka	−0.03 (–0.13 to 0.07)	−0.12 (–0.19 to −0.05)	−2.83 (–3.38 to −2.28)	−1.5 (–1.64 to −1.36)
Sudan	0.29 (0.2 to 0.37)	−0.03 (–0.08 to 0.03)	−1.9 (–2.11 to −1.7)	−1.31 (–1.41 to −1.2)
Suriname	0.28 (0.26 to 0.3)	0.01 (–0.04 to 0.07)	−1.2 (–1.88 to −0.52)	−0.75 (–0.99 to −0.51)
Sweden	−0.26 (–0.33 to −0.19)	−0.24 (–0.3 to −0.19)	−1.45 (–2.73 to −0.16)	−0.83 (–1.05 to −0.62)
Switzerland	0.04 (–0.05 to 0.12)	−0.2 (–0.27 to −0.14)	−0.77 (–1.1 to −0.44)	−0.64 (–0.81 to −0.48)
Syrian Arab Republic	0.35 (0.17 to 0.53)	0.44 (0.27 to 0.6)	−0.89 (–1.16 to −0.62)	−0.57 (–0.78 to −0.36)
Taiwan (Province of China)	0.27 (0.19 to 0.36)	0.15 (0.08 to 0.21)	−0.86 (–2 to 0.29)	−0.61 (–1.08 to −0.14)
Tajikistan	−0.62 (–0.75 to −0.49)	−0.91 (–0.99 to −0.83)	−0.17 (–0.71 to 0.37)	−0.59 (–1.04 to −0.13)
Thailand	0.63 (0.59 to 0.66)	0.86 (0.83 to 0.88)	−1.15 (–1.4 to −0.89)	−0.24 (–0.33 to −0.15)
Timor–Leste	0.39 (0.18 to 0.61)	0.26 (0.02 to 0.5)	−1.07 (–1.44 to −0.69)	−0.31 (–0.5 to −0.13)
Togo	−0.72 (–0.86 to −0.58)	−1.16 (–1.32 to −1)	−0.36 (–0.79 to 0.07)	−1.23 (–1.38 to −1.09)
Tokelau	0.14 (0.01 to 0.28)	0.08 (–0.05 to 0.22)	4.69 (3.74 to 5.65)	2.06 (1.03 to 3.09)
Tonga	0.2 (0.15 to 0.25)	0.23 (0.2 to 0.25)	−0.19 (–0.52 to 0.14)	−0.03 (–0.17 to 0.1)
Trinidad and Tobago	0.11 (0 to 0.21)	0 (–0.16 to 0.15)	−1.99 (–3.42 to −0.54)	−0.98 (–1.38 to −0.57)
Tunisia	0.53 (0.46 to 0.59)	0.04 (–0.02 to 0.11)	−2.95 (–3.11 to −2.78)	−1.72 (–1.84 to −1.61)
Turkey	0.54 (0.41 to 0.67)	−0.32 (–0.58 to −0.06)	−2.88 (–3.31 to −2.44)	−2.12 (–2.41 to −1.83)
Turkmenistan	0.31 (0.17 to 0.46)	0.31 (0.25 to 0.37)	0.76 (0.6 to 0.92)	0.31 (0.12 to 0.5)
Tuvalu	0.03 (–0.08 to 0.13)	−0.06 (–0.15 to 0.03)	−0.75 (–0.93 to −0.57)	−0.59 (–0.71 to −0.48)
Uganda	−0.07 (–0.21 to 0.07)	−0.22 (–0.34 to −0.1)	0.02 (–0.36 to 0.41)	−0.21 (–0.55 to 0.14)
Ukraine	−0.54 (–0.61 to −0.47)	−0.6 (–0.65 to −0.55)	−2.18 (–3.49 to −0.86)	−1.39 (–1.83 to −0.94)
United Arab Emirates	−0.23 (–0.31 to −0.15)	−0.89 (–0.97 to −0.82)	−4.2 (–5.89 to −2.47)	−2.07 (–2.37 to −1.77)
United Kingdom	0.21 (0.12 to 0.3)	0.19 (0.09 to 0.28)	−2 (–3.05 to −0.95)	−0.84 (–1.02 to −0.67)
United Republic of Tanzania	0.49 (0.13 to 0.85)	0.52 (0.15 to 0.9)	−0.62 (–0.86 to −0.38)	−0.3 (–0.49 to −0.1)
United States of America	0.21 (0.13 to 0.29)	0.11 (–0.01 to 0.23)	0.67 (0.07 to 1.28)	0.04 (–0.08 to 0.15)
United States Virgin Islands	−0.24 (–0.35 to −0.13)	−0.28 (–0.43 to −0.13)	−1.31 (–3.23 to 0.64)	−0.88 (–1.3 to −0.45)
Uruguay	0.39 (0.36 to 0.43)	0.04 (0.02 to 0.06)	−0.32 (–0.55 to −0.1)	−0.45 (–0.64 to −0.27)
Uzbekistan	0.21 (0.01 to 0.4)	0.19 (0.11 to 0.27)	−0.04 (–0.45 to 0.38)	−0.16 (–0.4 to 0.07)
Vanuatu	0.13 (0.11 to 0.15)	0.14 (0.09 to 0.18)	0.37 (–0.2 to 0.95)	0.17 (–0.03 to 0.37)
Venezuela (Bolivarian Republic of)	−0.16 (–0.21 to −0.11)	−0.28 (–0.33 to −0.23)	−0.07 (–1.32 to 1.19)	−0.55 (–0.81 to −0.3)
Viet Nam	0.72 (0.53 to 0.9)	0.88 (0.78 to 0.99)	−0.67 (–0.83 to −0.52)	0.22 (0.11 to 0.34)
Yemen	0.26 (0.19 to 0.33)	−0.31 (–0.37 to −0.24)	−1.14 (–1.59 to −0.69)	−0.97 (–1.15 to −0.78)
Zambia	0.18 (0.08 to 0.28)	−0.02 (–0.14 to 0.1)	0.58 (0.26 to 0.9)	0.14 (–0.16 to 0.44)
Zimbabwe	0.07 (0.02 to 0.11)	−0.05 (–0.13 to 0.02)	1.18 (–0.28 to 2.66)	0.45 (0.06 to 0.84)

### Decomposition analysis

**Figure 5.**
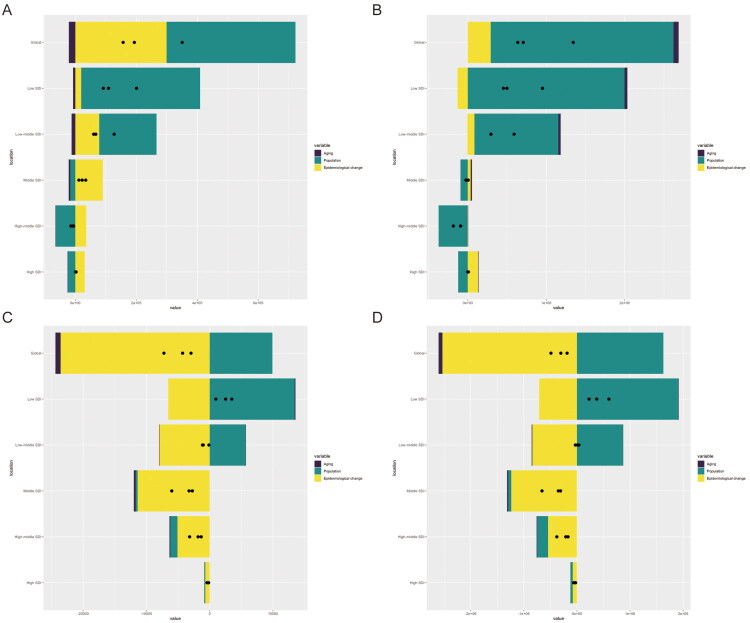
The relative contributions of aging, population, and epidemiological changes to the number of incidence (a), prevalence (B), mortality (C) and DALYs (D) from 1990 to 2021, comparison in the world, and 5 SDI regions. The black dot represents the overall difference of incidence and mortality from 1990 to 2021.

As shown in Figure 5, the global increase in incidence and prevalence of epilepsy in children and adolescents is primarily attributed to population factors, with a lesser contribution from epidemiological changes. In contrast, the decrease in mortality and DALYs is mainly driven by epidemiological changes. In the five major SDI regions, the increase in incidence and prevalence in the low and middle-low SDI regions is largely due to population changes, while declines in mortality and DALYs are attributed to epidemiological factors. The middle SDI region showed an increase in incidence due to population changes, a slight decrease in prevalence, and significant declines in mortality and DALYs, which were attributed to epidemiological changes. In the high-middle SDI region, all four indicators showed declines. Incidence and prevalence were primarily driven by population changes, while reductions in mortality and DALYs were due to epidemiological factors. The high SDI region exhibited no significant changes in any of the four indicators.

### Disease burden prediction

**Figure 6.**
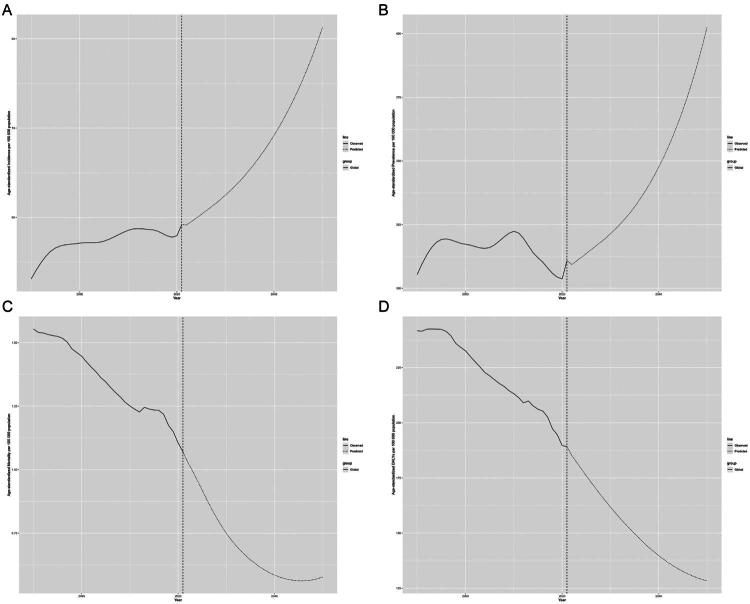
Prediction of change in the burden of epilepsy in children and adolescents to 2050 (A) age-standardized incidence; (B) age-standardized prevalence; (C) age-standardized mortality; (D) age-standardized DALYs.

The future disease burden of epilepsy in children and adolescents was projected using Bayesian spatiotemporal modeling. Both incidence and prevalence are expected to continue rising, while mortality and DALYs are projected to decline. Global incidence is forecasted to reach 81.3/100,000 population by 2050, reflecting a 37.3% increase, while prevalence is expected to rise to 402.6/100,000 population, a 32.2% increase. Mortality is projected to decrease to 0.6/100,000 population, and DALYs are expected to decrease to 128.3/100,000 population (Figure 6).

## Discussion

### Main interpretation

This study assessed the global disease burden of epilepsy in children and adolescents, which remains high overall, particularly in low-income countries. In these regions, due to national and social constraints, the low level of awareness and public acceptance of epilepsy directly contributes to the increased disease burden. Between 1990 and 2021, incidence showed an increasing trend, while prevalence did not exhibit significant changes. Mortality and DALYs demonstrated a clear downward trend. The rising incidence of epilepsy among children and adolescents may be attributed to multiple factors, including improvements in diagnostic techniques and the increased availability of screening programs. Meanwhile, advances in medical technology—such as the development of treatment plans based on electroencephalogram (EEG) findings—and improved access to antiepileptic drugs have contributed to the marked decline in epilepsy-related mortality and DALYs [3,27,28]. We further examined the disease burden across 21 regions. The results revealed that epilepsy was most prevalent in children under 5 years of age, with higher prevalence rates in the 15–19 and 10–14 age groups. The distribution of mortality and DALYs varied significantly by region but was predominantly concentrated in the 15–19 age group in most regions. As seen in previous studies, males exhibited higher prevalence and disease burden in most regions [23,29]. Overall, East Asian countries exhibited the lowest disease burden. Among the 204 countries, most showed a downward trend in epilepsy burden, with the most notable decline observed in China. In recent years, low-SDI countries have improved access to existing treatments and developed new therapeutic agents. Additionally, the United Nations has made the reduction of the burden of neurological diseases a key component of its Sustainable Development Goals. These initiatives have contributed to a reduction in the disease burden of epilepsy, aligning with the results of our decomposition analysis [5,21]. Furthermore, countries with lower SDI exhibit a higher disease burden, which is consistent with our findings [2,20,22]. This suggests that health resources should be appropriately allocated to lower SDI regions to reduce the global disease burden.

In low-SDI regions, national, social, economic, and cultural constraints hinder awareness and acceptance of epilepsy. Additionally, the lack of local resources relative to population size, delayed treatment, lack of awareness, and ineffective screening and prevention efforts contribute to the disease burden [2,18]^–^ [22]. Inadequate prenatal screening, perinatal hypoxia, congenital brain malformations, and cortical dysplasia contribute to the increased incidence of epilepsy in children in low-income regions [11,30]. In low-SDI regions, parasitic infections such as cerebral malaria, Taenia solium cysticercosis and onchocerciasis are important etiological factors contributing to the development of epilepsy [31]. *Onchocerca volvulus*, a parasitic worm endemic to several African countries such as South Sudan and Cameroon, is associated with onchocerciasis-associated epilepsy (OAE), which predominantly affects children and adolescents aged 3–18 years. This condition imposes a significant disease burden on affected communities [9]. In more developed regions, governments place greater emphasis on epilepsy awareness, screening, and prevention, and treatments are more diverse and widely accessible [20,32]. These efforts have helped reduce the burden of epilepsy.

In recent years, numerous global efforts have been undertaken to reduce the burden of epilepsy. As early as 1997, the World Health Organization (WHO) launched the Global Campaign Against Epilepsy. In China, this initiative led to the implementation of physician training programs for epilepsy diagnosis and treatment in rural areas across six provinces, significantly alleviating the disease burden. In 2015, the WHO adopted a resolution urging member states to implement preventive interventions and emphasized the need for all individuals with epilepsy to have access to high-quality, affordable treatment. In 2019, Russia identified epilepsy as a national health priority. Most recently, in 2022, the WHO adopted the Intersectoral Global Action Plan on Epilepsy and Other Neurological Disorders, which aims to improve access to care and address stigma associated with these conditions [33]. In low-SDI regions multiple initiatives have aimed to reduce the burden of epilepsy. For example, the WHO launched the Expanded Special Project for the Elimination of Neglected Tropical Diseases (ESPEN) in Africa and established the Global Onchocerciasis Network for Elimination to coordinate efforts against onchocerciasis [9,28]. Although the disease burden of epilepsy among children and adolescents has been effectively alleviated in recent years, increasing public awareness and improving early screening should remain priority actions. Governments are encouraged to allocate more resources to public health. In addition, high-income countries should strengthen international collaboration and promote data sharing to support global efforts in reducing the epilepsy burden [2,18]. Treatment facilities, such as EEG, and access to medications are limited in low-SDI regions. International public health organizations should focus on improving access to drugs, medical equipment, and trained healthcare professionals in these areas [21]. Advanced resource planning and policy development, based on projections of future disease burden, can effectively reduce the epilepsy burden in children and adolescents.

In recent years, research has increasingly focused on alterations in epileptic EEG[34,35], gene mutations, inflammatory responses, lipid peroxidation, ion channels, and their role in epilepsy [10,36–38]. However, many underlying mechanisms remain unclear. Further exploration of the molecular mechanisms of epilepsy is essential to provide new insights into early screening and personalized treatment options.

### Limitation

This study has several limitations. First, some of the data for certain countries were estimated using statistical modeling, which may introduce discrepancies from the actual values. Second, as a secondary analysis, although we included global data, the wide range of UIs for some regions in the raw data highlights the heterogeneity of the data sources [5]. Moreover, the limited availability or complete absence of data in certain countries may have influenced the outcomes of our study. To address these limitations, more comprehensive and higher-quality primary data are needed. Third, this study was limited to individuals under 20 years of age, and the impact of epilepsy on the broader population remains unclear.

## Conclusion

Epilepsy is a major neurological disorder, commonly linked with anxiety, depression, and neurocognitive impairment, contributing significantly to the global disease burden, particularly among children and adolescents. While the burden of epilepsy in children and adolescents has decreased in recent years, it still represents one of the highest disease burdens globally. Population factors are the primary contributors to the increase in disease burden, while epidemiological factors, including favorable public health policies and improved access to treatments, have played a key role in reducing the burden. The future burden of epilepsy will remain substantial. To mitigate this, improvements in treatment access, increased support for countries with low SDIs, and adjustments in health budget allocations will be essential. Additionally, the formulation and optimization of personalized policies are essential measures for reducing the burden of epilepsy. Governments, research institutions, and international organizations must collaborate closely to address the burden of epilepsy.

## Data Availability

All data used in this study can be find at the GBD 2021 study (https://vizhub.healthdata.org/gbd-results). Further data can be requested from the corresponding author.
